# An Orthotopic Xenograft Model for Preclinical Evaluation of CAR T Cell Therapy in Glioblastoma

**DOI:** 10.1002/cpz1.70503

**Published:** 2026-10-30

**Authors:** Julian Hübner, Rhonda McFleder, Chi Wang Ip, Miriam Alb, Hermann Einsele, Michael Hudecek, Thomas Nerreter

**Affiliations:** ^1^ Chair of Cellular Immunotherapy, Department of Medicine II University Hospital Würzburg Würzburg Germany; ^2^ Department of Neurology University Hospital Würzburg Würzburg Germany; ^3^ Department of Medicine II University Hospital Würzburg Würzburg Germany

**Keywords:** bioluminescence, CAR T cells, flow cytometry, glioblastoma, stereotactic surgery

## Abstract

Glioblastoma (GBM) is the most lethal primary brain tumor in adults, and despite intensive research efforts, effective therapeutic breakthroughs remain elusive. Consequently, the standard of care has remained largely unchanged for nearly 30 years, underscoring the need for robust preclinical models to evaluate experimental therapies. One particularly promising approach is chimeric antigen receptor (CAR) T cell therapy, which has demonstrated remarkable success in several hematologic malignancies but has not yet achieved comparable efficacy in solid tumors. This article describes the generation of orthotopic GBM xenografts in NSG mice, including anesthesia, surgical procedures, and postoperative care, followed by locoregional delivery of human CAR T cells into the lateral ventricle and subsequent assessment of therapeutic efficacy. Together, these protocols provide a reliable platform for evaluating GBM‐directed CAR T cell therapies in both basic and translational research. © 2026 The Author(s). *Current Protocols* published by Wiley Periodicals LLC.

**Basic Protocol 1**: Intracranial tumor implantation

**Basic Protocol 2**: Intraventricular CAR T cell administration

**Support Protocol 1**: Bioluminescence imaging of xenografts

**Support Protocol 2**: Endpoint analysis of residual tumor burden by flow cytometry

## INTRODUCTION

Glioblastoma (GBM) is the most common and aggressive primary malignant brain tumor in adults and remains largely refractory to current multimodal treatment approaches (Marton et al., 2020; Thakkar et al., 2014). Although chimeric antigen receptor (CAR) T cell therapies have demonstrated remarkable clinical efficacy in CD19‐positive hematologic malignancies, similar therapeutic success has not yet been achieved in solid tumors, particularly in GBM (Cappell & Kochenderfer, 2023; June & Sadelain, 2018; Rodríguez‐Lobato et al., 2020). The limited efficacy observed in GBM can be attributed, in part, to inefficient trafficking of CAR T cells to intracranial tumor lesions and substantial inter‐ and intratumoral antigen heterogeneity (Bagley & O'Rourke, 2020; Hou et al., 2021).

In response to these challenges, locoregional administration of CAR T cells targeting novel antigens, multimodal treatment strategies, and combinations of immunotherapeutic approaches aimed at overcoming tumor heterogeneity are currently being investigated in the field of CAR T cell therapy for GBM (Cao et al., 2025; Sterner & Sterner, 2021; Zhu et al., 2025). Intrathecal administration of CAR T cells via an Ommaya reservoir has been shown to achieve higher intratumoral concentrations while reducing systemic exposure compared with intravenous delivery (Geurts & Preusser, 2024). A robust and reproducible preclinical platform that enables systematic evaluation of CAR T cell efficacy in orthotopic tumor models is critically needed to assess therapeutic efficacy accurately and reproducibly.

Here, we describe a set of protocols for establishing orthotopic xenografts in NSG mice (Basic Protocol 1) and subsequent locoregional administration of CAR T cells into the lateral ventricle to treat engrafted tumors (Basic Protocol 2). *In vivo* bioluminescence imaging (BLI; Support Protocol 1) can be used to confirm successful tumor engraftment and to monitor and quantify tumor growth and regression over time. In addition, endpoint assessment of residual tumor burden in mouse brains can be performed by flow cytometry (Support Protocol 2), enabling precise determination of treatment response.

*NOTE*: All protocols involving animals must be reviewed and approved by the appropriate Animal Care and Use Committee and must follow regulations for the care and use of laboratory animals.

## Basic Protocol 1 INTRACRANIAL TUMOR IMPLANTATION

This protocol describes the orthotopic implantation of GBM cells into the brain of NSG mice via stereotactic surgery.

### Materials

Firefly luciferase/enhanced green fluorescent protein (ffluc/eGFP)‐expressing GBM cells
GBM medium (see recipe), 37°C
Dulbecco's phosphate‐buffered saline (DPBS), no calcium, no magnesium (Thermo Fisher, 14190169)
Accutase® Cell Detachment Solution (BioLegend, 423201)
0.9% (w/v) NaCl infusion solution (e.g., B. Braun, 6726174.00.00), sterile, 4°C
Mice: female 6‐ to 8‐week‐old NSG mice (NOD.Cg‐Prkdcscid Il2rgtm1Wjl/SzJ, Charles River Laboratories, JAX stock no. 005557).
Isoflurane, 1000 mg/g
Carprofen, 50 mg/ml
Lubricating ointment (e.g., Bepanthen^®^ Augen‐ und Nasensalbe 5% Dexpanthenol, Bayer, 01578681)
70% (v/v) ethanol and/or similar disinfectant
Alcohol‐based skin antiseptic (e.g., Octeniderm, Schülke, 17938986)
UltraPure^TM^ distilled water (Thermo Fisher, 10977‐015)
Bone wax (Ethicon, W31C)
Antibiotic ointment (e.g., Fucidine 20 mg/g, Leo Pharma A/S, 6424177.00.01)

T‐75 flasks
15‐ml conical tubes
Refrigerated centrifuge, 4°C
1.5‐ml reaction tubes, sterile
Induction chamber (Xenogen XGI‐8 Gas Anesthesia System)
Stereotactic system, including head holder with tooth bar and ear bars, anesthesia mask, frame, arm, drill, and injection pump (part of microinjection robot; Neurostar)
U‐100 insulin syringes (100‐IU capacity; e.g., Omnican^®^ 100 with 30G needles, B. Braun, 00465667)
Rodent warmer, with heating pad and rectal temperature probe (Stoelting, 53800M and 53805)
Small‐animal trimmer
Cotton‐tipped applicators, sterile
Surgical scissors, sterile
Forceps, sterile
Stereomicroscope (Zeiss, Stemi 508)
10‐µl Hamilton® syringe [model no. 701N, cemented needle, needle size 26s ga (bevel tip), needle L 51 mm (2 in.), Sigma‐Aldrich, 20779]
Stereotactic atlas (e.g., Gaidicia, n.d.; Paxinos & Franklin, 2001)
Autoclavable non‐toxic marker, sterile
0.8‐mm cylinder drill bit (Meisinger, 2100031104008), sterile
Coated Vicryl^TM^ Polyglactin 910 sutures (Ethicon, V797D)
Hemostats, sterile
Clean recovery cages placed on heating pad

Additional reagents and equipment for counting cells and for BLI (see Support Protocol 1)

*NOTE*: For this protocol, GBM cell lines stably transduced to express ffluc and eGFP under the control of an EF1α‐HTLV fusion promoter are used. Any commercially available or laboratory‐generated GBM cell line stably expressing ffluc and eGFP may be used.

*NOTE*: Established GBM cell lines maintained under standard adherent culture conditions using GBM medium (see recipe) are used in this protocol to generate orthotopic xenografts. It is strongly recommended to expand GBM cells before initiating animal experiments and to cryopreserve large batches at the same passage number. This ensures consistent use of low‐passage cells throughout the study. Thaw cells ∼2 weeks before implantation and subculture them 2 to 3 times per week once they reach 70% to 80% confluence to maintain sub‐confluent cultures. Harvest cells during the logarithmic growth phase. Consistent cell preparation is essential for reproducibility and data quality.

*NOTE*: All solutions and equipment coming into contact with cells must be sterile, and proper sterile technique should be used accordingly.

*NOTE*: All culture incubations are performed in a 37°C, 5% CO_2_ incubator unless otherwise specified.

*NOTE*: The use of patient‐derived GBM models, alternative culture conditions (e.g., serum‐free or factor‐defined medium), extracellular matrix coatings, or different culture formats (e.g., adherent versus neurosphere cultures) may influence the phenotype, tumorigenicity, and growth kinetics of the cells following intracranial implantation. Accordingly, if alternative GBM models or culture conditions are used, the parameters outlined in this protocol should be considered a starting point and should be validated and, where necessary, optimized in preliminary experiments.

*NOTE*: Sterilize all surgical instruments before use. Instruments requiring sterilization include the drill bit, forceps, hemostats, and surgical scissors. In addition, researchers should wear a sterile gown, gloves, hairnet, and mask throughout the animal procedures.

#### Culture and preparation of GBM cells for stereotactic injection

1 Culture ffluc/eGFP‐expressing GBM cells in 20 ml GBM medium in T‐75 flasks.
  The number of flasks required depends on the number of mice to be injected. This protocol uses 2 × 10^5^ cells per mouse. Prepare an additional 25% to 50% to compensate for dead volume and potential surgical losses.
2 After ∼2 weeks of culture, when cells reach ∼80% confluence, wash once with DPBS and detach the cells using Accutase® Cell Detachment Solution (2 ml per T‐75 flask; <5 min at 37°C).
3 Neutralize the Accutase by adding pre‐warmed GBM medium at four times the volume of Accutase used. Collect the cells in 15‐ml conical tubes and centrifuge for 6 min at ∼300 × *g*, 4°C.
4 Resuspend the cell pellet in 10 ml DPBS, determine the cell count, and confirm a viability of >90%.
5 Resuspend the cells in ice‐cold sterile 0.9% NaCl infusion solution at the desired concentration (e.g., 4 × 10^7^ cells/ml to inject 2 × 10^5^ cells in 5 µl per mouse) and transfer to sterile 1.5‐ml reaction tubes.
6 Keep the cell suspension on ice before and throughout stereotactic surgery.
  This helps to preserve viability and minimize cell aggregation, which may clog the Hamilton^®^ syringe (see step 23) and increase the risk of reflux during injection.

#### Preparation of mice for surgery

7 Place the mouse in an induction chamber and anesthetize with 2% to 4% isoflurane.
  Surgical anesthesia is typically achieved after ∼5 min.
8 Secure the incisors in the tooth bar with the tongue positioned beneath the bar. Position the stereotactic anesthesia mask over the nose and maintain anesthesia with 1.5% to 2% isoflurane.
9 Confirm an adequate surgical plane of anesthesia by verifying the absence of pedal withdrawal reflexes while continuously monitoring respiration.
10 Administer carprofen (5 mg/kg, subcutaneously) with U‐100 insulin syringe to provide preemptive analgesia.
  IMPORTANT NOTE: Administer carprofen ≥30 min before surgery to allow sufficient time for analgesic onset.
11 Lubricate the rectal temperature probe with lubricating ointment and insert it gently into the rectum. Adjust the heating pad to maintain a body temperature of approximately 32° to 33°C.
12 Position the ear bars carefully and secure the skull so that it is level and parallel to the base of the stereotactic frame.
  When correctly positioned, the head should move only in the dorsoventral direction and should not exhibit lateral or rotational movement.
13 Remove the fur from the scalp using a small‐animal trimmer.
14 Add lubricating ointment to both eyes to prevent corneal drying.
15 Disinfect gloved hands with 70% ethanol or similar disinfectant to maintain aseptic technique.
  Repeat this disinfection regularly throughout the procedure.
16 Disinfect the surgical field using a sterile cotton‐tipped applicator moistened with alcohol‐based skin antiseptic.
17 Thirty minutes after carprofen administration (see step 10), create a small parasagittal skin incision using sterile surgical scissors.
18 Retract the skin with sterile forceps to expose the skull. Clean the skull surface with a sterile cotton‐tipped applicator moistened with antiseptic to clearly identify the stereotactic landmarks Bregma and Lambda.

#### Stereotactic injection

19 Using a stereomicroscope, position the stereotactic arm so that the needle of the 10‐µl Hamilton® syringe is centered directly above Bregma.
  Use of a wall‐mounted surgical microscope is strongly recommended to maximize stereotactic accuracy.
  Use Bregma as the origin for all stereotactic coordinates. Before proceeding, level the skull by aligning Bregma and Lambda within the same dorsoventral plane (see Fig. 1). The difference between these landmarks should not exceed 0.03 mm on the dorsoventral axis. No adjustment of the anteroposterior or mediolateral coordinates should be required when moving between Bregma and Lambda. If such adjustments are necessary, reposition the ear bars before continuing.

**Figure 1:**
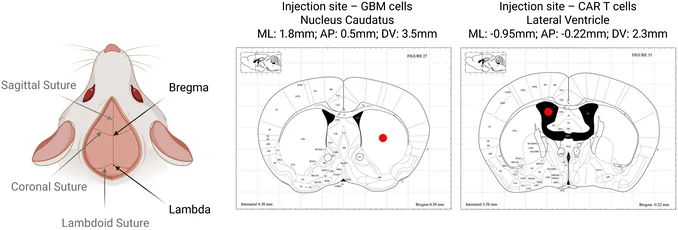
A midline scalp incision exposes the anatomy of the mouse skull (left). Bregma is identified as the intersection of the sagittal suture with the coronal suture. Lambda is located at the posterior end of the sagittal suture, immediately rostral to the apex of the lambdoid suture, corresponding to the point where the sagittal suture would intersect a best‐fit line through the lambdoid suture. These anatomical landmarks are used to calibrate the stereotactic frame, enabling precise targeting of the caudate nucleus for tumor cell implantation (center) and the lateral ventricle for intraventricular CAR T cell administration (right) using the corresponding stereotactic coordinates.

20 Move the needle of the 10‐µl Hamilton® syringe to the desired target coordinates using a stereotactic atlas. Mark the entry point on the skull with a sterile autoclavable non‐toxic marker.
21 Drill a small burr hole through the skull using a sterile 0.8‐mm cylinder drill bit. Remove any blood using a sterile cotton‐tipped applicator before proceeding.
  Advance the drill slowly, without excessive pressure.
22 Clean the 10‐µl Hamilton^®^ syringe thoroughly by flushing it with 70% ethanol, followed by multiple rinses with UltraPure^TM^ distilled water, and then air‐dry completely.
23 Resuspend the cell suspension from step 6 immediately before loading and then draw 5 µl into the 10‐µl Hamilton^®^ syringe.
24 Mount the syringe in the injection pump and position the needle above the burr hole using the predetermined coordinates (ML: 1.8 mm; AP: 0.5 mm; see Fig. 1). Advance the needle to a depth of 3.8 mm (DV) at 0.1 mm/s. After 1 min, retract the needle to 3.5 mm (DV) at the same speed to create a small cavity for the cell suspension.
25 Inject 5 µl of the cell suspension at 1 µl/min. After completion of the injection, leave the needle in place for an additional 1 min.
  Creating a small cavity before injection and injecting at a slow rate minimize tissue trauma and reduce reflux through the burr hole, thereby improving consistency of tumor engraftment.
26 Withdraw the needle slowly, at 1 mm/min, to minimize reflux.
27 Clean the Hamilton^®^ syringe immediately by repeatedly flushing it with distilled water.
28 Seal the burr hole with bone wax and close the incision with coated Vicryl™ Polyglactin 910 sutures using sterile hemostats and forceps. Apply 3 to 4 interrupted sutures, depending on incision length, and cover the wound with antibiotic ointment.
29 Remove the ear bars and anesthesia mask and then transfer the mouse carefully to a clean recovery cage placed on a heating pad.
30 Monitor the mouse continuously until full recovery from anesthesia. Once normal ambulation and activity have resumed, return the cage to the animal housing room.
31 Administer carprofen (5 mg/kg, subcutaneously) every 24 hr for the following 48 hr to provide postoperative analgesia.
32 Monitor animals daily throughout the experiment according to the approved animal protocol, as neurological deterioration can occur rapidly.
  Common signs include hunched posture, circling behavior, toe walking, impaired balance, closed eyes, weight loss, hyperactivity, and seizures.
33 Perform BLI at the predefined experimental time points to confirm successful tumor engraftment and monitor tumor progression (see Support Protocol 1).

## Basic Protocol 2 INTRAVENTRICULAR CAR T CELL ADMINISTRATION

This protocol describes the stereotactic administration of CAR T cells into the brains of NSG mice previously engrafted with orthotopic GBM xenografts (Basic Protocol 1). CAR T cells are injected into the lateral ventricle of the hemisphere contralateral to the tumor, allowing them to distribute throughout the cerebrospinal fluid and migrate toward the tumor site.

### Additional Materials (also see Basic Protocol 1)

CAR T cells
CAR T cell medium (see recipe), 37°C
Tumor‐bearing mice from Basic Protocol 1

Additional reagents and equipment for generating and expanding CAR T cells (see Current Protocols article: Prommersberger et al., 2020) and endpoint analysis of residual tumor burden by flow cytometry (see Support Protocol 2)

*NOTE*: CAR T cells may be generated using viral, non‐viral integrating, transient, or site‐specific genome‐editing approaches. Together with the overall culture conditions and manufacturing protocol, these approaches may substantially influence the resulting phenotype and functional activity of the CAR T cell product. In combination with differences in the tumorigenicity and *in vivo* growth kinetics of individual tumor cell lines or patient‐derived models, these factors can significantly affect the outcome of the model. Therefore, it is strongly recommended to evaluate both the tumorigenicity of the selected tumor cells and the antitumoral activity of the resulting CAR T cell product in preliminary experiments and to empirically optimize the experimental parameters accordingly. In this protocol, CAR T cells are administered 7 days after tumor implantation (Basic Protocol 1) at an effector‐to‐target (E:T) ratio of 10:1. The timing and dose of CAR T cell administration should be considered a starting point and may require adjustment depending on the tumor model, tumor burden, and potency of the cell product.

#### Generation of CAR T cells and preparation for stereotactic injection

1 Generate and expand CAR T cells in CAR T cell medium as previously described (see Current Protocols article: Prommersberger et al., 2020).
2 Harvest the required number of CAR T cells, confirm a viability of >90%, and wash the cells with DPBS.
  The number of cells required depends on the desired dose per mouse. In this protocol, an E ratio of 10:1 is achieved by administering 2 × 10^6^ CAR T cells per mouse. Prepare an additional 25% to 50% to compensate for dead volume and potential surgical losses.
3 Resuspend the cells in ice‐cold sterile 0.9% NaCl infusion solution at the desired concentration (e.g., 4 × 10^8^ cells/ml to inject 2 × 10^6^ cells in 5 µl per mouse).
4 Keep the cell suspension on ice throughout the surgical procedure to preserve cell viability and minimize cell aggregation, which may clog the Hamilton^®^ syringe (see step 11) and increase the risk of reflux during injection.

#### Preparation of mice for surgery

5 Prepare tumor‐bearing mice from Basic Protocol 1 for surgery as described in Basic Protocol 1, steps 7 to 12.
6 Apply lubricating ointment to both eyes to prevent corneal drying.
7 Disinfect gloved hands regularly throughout the procedure with 70% ethanol or similar disinfectant to maintain aseptic technique.
8 Disinfect the skin overlying the previous surgical incision using a sterile cotton‐tipped applicator moistened with alcohol‐based skin antiseptic.
9 Thirty minutes after carprofen administration, carefully reopen the parasagittal incision using sterile surgical scissors.
10 Retract the skin to expose the skull. Clean the skull surface with a sterile cotton‐tipped applicator moistened with antiseptic to identify the stereotactic landmarks Bregma and Lambda (see Fig. 1).
  Owing to wound healing and tissue remodeling, the skull surface may differ from that observed during the initial surgery (see Basic Protocol 1), potentially making identification of Bregma and Lambda more challenging and affecting drilling characteristics. Familiarity with this procedure is recommended to ensure stereotactic accuracy and experimental reproducibility.

#### Stereotactic injection

11 Perform stereotactic needle positioning as described in Basic Protocol 1, steps 19 to 24, using the coordinates for intraventricular CAR T cell administration (ML: 0.95 mm; AP: 0.22 mm; DV: 2.3 mm; see Fig. 1). Advance the needle at a rate of 0.1 mm/s.
12 Inject 5 µl of the CAR T cell suspension from step 4 at a rate of 1 µl/min and leave the needle in place for an additional 1 min before withdrawal.
13 Complete the surgical procedure as described in Basic Protocol 1, steps 26 to 32. Monitor tumor regression by BLI at the predefined experimental time points (see Support Protocol 1).
14 As an additional endpoint analysis, determine residual tumor burden by flow cytometry analysis of mouse brain tissue as described in Support Protocol 2.

## Support Protocol 1 BIOLUMINESCENCE IMAGING OF XENOGRAFTS

This protocol describes the use of *in vivo* bioluminescence imaging (BLI) to monitor orthotopic GBM xenografts established using Basic Protocols 1 and 2. BLI enables confirmation of successful tumor implantation before treatment (Basic Protocol 1), facilitates randomization of experimental groups based on tumor burden (following Basic Protocol 1), and allows longitudinal monitoring of tumor progression or regression following CAR T cell therapy (Basic Protocol 2). In addition, BLI can serve as an objective measure of disease progression and may be incorporated into predefined humane endpoint criteria to minimize animal burden.

### Materials

D‐luciferin solution (see recipe)
Mice from Basic Protocol 1 or 2
Isoflurane, 1000 mg/g

IVIS *in vivo* imaging system (IVIS Lumina XRMS Series III , Revvity Health Sciences) and associated computer
Living Image software (Revvity Health Sciences)
Isoflurane vaporizer, including induction chamber and nose cone (Xenogen XGI‐8 Gas Anesthesia System)
U‐100 insulin syringes (100‐IU capacity; e.g., Omnican^®^ 100 with 30G needles, B. Braun, 00465667)

*NOTE*: The protocol describes BLI using an IVIS Lumina imaging system and Living Image software (Revvity Health Sciences). Imaging parameters and software operation may differ between instrument models and software versions. Users should consult the corresponding manufacturer's documentation where appropriate.

1 Turn on the IVIS *in vivo* imaging system and the associated computer.
2 Launch Living Image software, log in, and initialize the imaging system. Wait until the CCD camera has cooled to −90°C before acquiring images.
3 Administer D‐luciferin solution to a mouse from Basic Protocol 1 or 2 intraperitoneally at 250 mg/kg in a total volume of 200 µl with U‐100 insulin syringe.
4 Ten minutes after D‐luciferin administration, anesthetize the mouse with 2% to 4% isoflurane in an induction chamber. Once an adequate depth of anesthesia has been reached (typically after ∼5 min), transfer the animal to the imaging chamber.
5 Position the mouse in the IVIS imaging chamber in the prone position and maintain anesthesia with ∼1.5% isoflurane delivered through the nose cone throughout image acquisition.
6 Close the door of the imager and maintain anesthesia with 1.5% isoflurane during the imaging process.
7 Acquire and analyze images as described in a Current Protocols article by Yamada‐Hara et al. (2024; Support Protocol, steps 10 to 32) or according to the standard operating procedure established for the respective IVIS imaging system.
  Tumor burden should be assessed immediately before (following Basic Protocol 1) CAR T cell administration (see Basic Protocol 2) to confirm successful tumor establishment and enable randomization of treatment groups. Subsequently, BLI should be performed at predefined intervals throughout the study to monitor treatment response and support assessment of humane endpoints (see Fig. 2).

**Figure 2:**
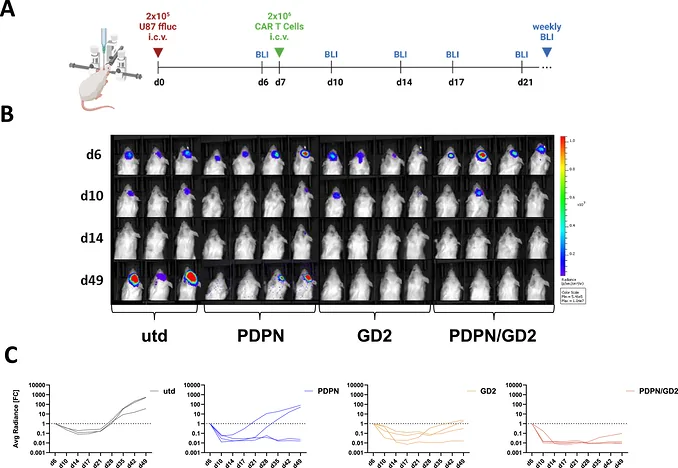
*In vivo* BLI enables longitudinal monitoring of tumor progression and regression. (**A**) Experimental timeline showing the time points of stereotactic tumor implantation, CAR T cell administration, and BLI. A total of 2 × 10^5^ U87 cells were stereotactically implanted into the brains of 6‐ to 8‐week‐old female NSG mice. Following confirmation of tumor establishment, 2 × 10^6^ untransduced (utd) control T cells or CAR T cells targeting PDPN, GD2, or both antigens (PDPN/GD2) were administered intraventricularly on day 7. Tumor burden was subsequently monitored by serial BLI. (**B**) Representative BLI images of the different treatment groups. (**C**) Quantification of the BLI signal, expressed as the fold change in average radiance over time for individual mice, demonstrates differential treatment responses among the experimental groups. Figure adapted from Hübner et al. (2026).

## Support Protocol 2 ENDPOINT ANALYSIS OF RESIDUAL TUMOR LOAD BY FLOW CYTOMETRY

This protocol describes the quantification of residual tumor burden in mouse brains by flow cytometry following euthanasia or at the experimental endpoint. Flow cytometric analysis enables sensitive detection of rare tumor cells within brain tissue at single‐cell resolution. The protocol outlines the preparation of single‐cell suspensions from mouse brain tissue and the subsequent quantification of eGFP‐positive tumor cells.

### Materials

Tumor‐bearing, control, and CAR T cell–treated mice (see Basic Protocol 2)
Enzymatic tissue dissociation kit (Tumor Dissociation Kit, human, Miltenyi Biotec, 130‐095‐929)
DPBS, no calcium, no magnesium (Thermo Fisher, 14190169)
ACK lysis buffer (ACK Lysing Buffer, Thermo Fisher, A1049201)
Debris removal solution (e.g., Miltenyi Biotec, 130‐109‐398)
Live/dead stain (e.g., Zombie Aqua™ Fixable Viability Kit, Thermo Fisher Scientific, 423102, or Ghost Dye Red710, Cytek Biosciences, 13‐0871‐T100)

CO_2_ inhalation/euthanasia system
Surgical scissors, sterile
Microspatula with spoon end, sterile
Scalpel, sterile
C tubes (Miltenyi Biotec, 130‐093‐237)
Tissue dissociator (gentleMACS^®^ Octo Dissociator with Heater, Miltenyi Biotec, 130‐134‐029)
70‐µm cell strainers (Easystrainer 70 µm, Greiner Bio‐One, cat. 542070)
15‐ and 50‐ml conical tubes
Refrigerated centrifuge, 4°C
Flow cytometer (e.g., BD FACSCanto™ II flow cytometer, Becton Dickinson, or Cytek^®^ Northern Lights™, Cytek Biosciences)
Flow data analysis software (e.g., FlowJo, Becton Dickinson)

1 Euthanize a tumor‐bearing, control, or CAR T cell–treated mouse by CO_2_ inhalation using 20% to 30% vol/min.
2 Decapitate the mouse using sterile surgical scissors and carefully dissect the brain from the skull. Remove surrounding muscle and connective tissue from the cranium to facilitate brain dissection.
3 Remove the skull by sequentially opening the occipital, temporal, and frontal regions using sterile surgical scissors. Carefully remove the brain using a sterile microspatula with a spoon end and excise the tissue surrounding the tumor implantation site using a sterile scalpel.
  Established GBM cell lines such as U87 typically form well‐demarcated tumors that can be dissected selectively. In contrast, patient‐derived xenografts and highly invasive GBM models often exhibit diffuse infiltration with poorly defined margins. In such cases, processing the entire brain for flow cytometric analysis is recommended to avoid underestimating tumor burden.
4 Dissociate the harvested brain tissue into a single‐cell suspension in C tubes using enzymatic and mechanical digestion with an enzymatic tissue dissociation kit and a tissue dissociator, respectively.
  Tissue dissociation can be performed using C tubes and the Miltenyi Tumor Dissociation Kit (human) according to the manufacturer's instructions and a gentleMACS^®^ Octo Dissociator using the program 37C_h_TDK_1.
5 Pass the cell suspension through a 70‐µm cell strainer placed on a 50‐ml conical tube.
6 Wash the cell strainer with 20 ml DPBS and centrifuge the cell suspension for 7 min at 300 × *g*, 4°C.
7 Resuspend the cell pellet in 1 ml ACK lysis buffer and incubate for 10 min at room temperature to lyse erythrocytes.
8 Stop the lysis by immediately adding 10 ml DPBS and then centrifuge the suspension for 10 min at 300 × *g*, 4°C.
9 Perform debris removal in 15‐ml conical tubes using debris removal solution.
  We use Miltenyi Debris Removal Solution according to the manufacturer's instructions.
10 Perform live/dead staining to exclude non‐viable cells prior to flow cytometric acquisition.
  Any viability dye may be used, provided that its emission spectrum does not overlap with the eGFP signal. When spectrally distinct fluorophores are selected, fluorescence compensation or spectral unmixing is generally not required.
11 Acquire samples on a flow cytometer and analyze the data using flow data analysis software by sequentially gating singlets, viable cells, and eGFP‐positive tumor cells.
  A representative gating strategy and representative analysis are shown in Figure 3.
  Endpoint flow cytometric analysis enables discrimination between mice that survived the predefined observation period and those that achieved sustained long‐term complete remission by confirming the absence of residual tumor cells (see Fig. 4).

**Figure 3:**
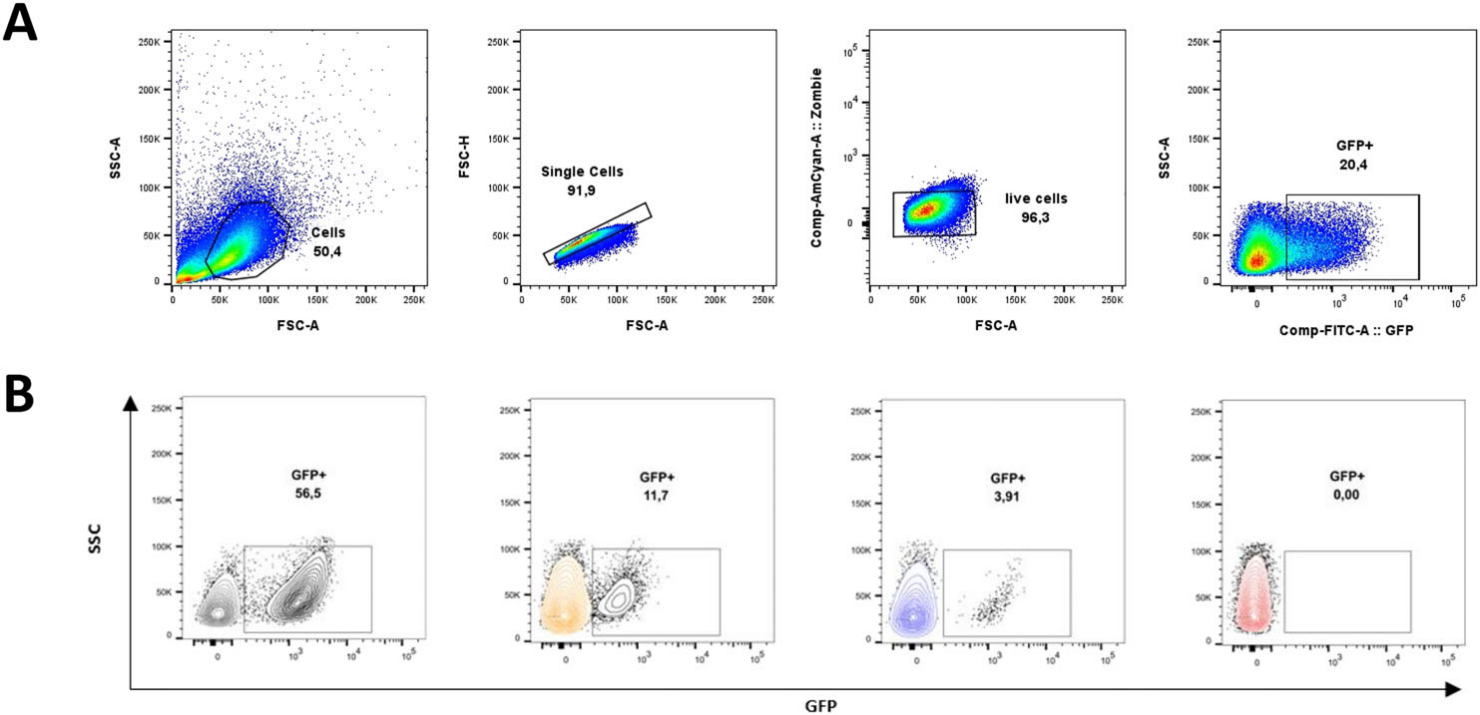
Flow cytometric analysis of residual tumor burden in mouse brains. (**A**) Representative gating strategy used to identify eGFP‐positive tumor cells by sequential gating of total cells, singlets, viable cells, and eGFP‐positive events. (**B**) Representative flow cytometric analysis of mouse brain tissue collected at the experimental endpoint. Figure adapted from Hübner et al. (2026).

**Figure 4:**
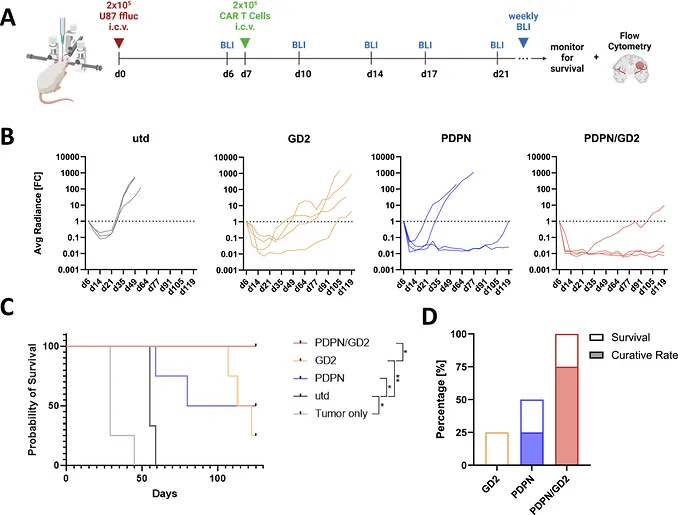
Combined *in vivo* BLI and endpoint flow cytometric analysis enable comprehensive evaluation of CAR T cell treatment efficacy. (**A**) Experimental timeline indicating the time points of BLI and flow cytometric analysis in the orthotopic U87 GBM model. (**B**) Longitudinal BLI demonstrates reduced tumor burden in mice treated with CAR T cells compared with mice receiving utd T cells. Data are displayed as the fold change in average radiance over time for individual mice. (**C**) Kaplan‐Meier survival analysis of untreated, utd T cell–treated, and CAR T cell–treated mice demonstrates the survival benefit associated with CAR T cell therapy (*, p<0.5; **, p<0.01). (**D**) Flow cytometric analysis of mouse brains (see Fig. 3) at the experimental endpoint enables quantification of residual tumor burden and assessment of durable or complete treatment responses in surviving animals. Figure adapted from Hübner et al. (2026).

## REAGENTS AND SOLUTIONS

### CAR T cell medium

500 ml RPMI‐1640, +GlutaMax, +25 mM HEPES (Thermo Fisher, 72400054)
50 ml human serum, heat‐inactivated (Bavarian Red Cross Blood Donor Service)
5 ml penicillin/streptomycin (10,000 U/ml, Thermo Fisher, 15140‐122)
5 ml GlutaMAX Supplement (100X) (Thermo Fisher, 35050038)
0.5 ml β‐mercaptoethanol (50 mM, Thermo Fisher, 31350010)
Store ≤1 month at 4°C

### D‐luciferin solution

1 g D‐luciferin potassium salt (Biosynth, L‐8220)
33 ml sterile H_2_O for injection (Ampuwa^®^, Fresenius Kabi, 40676.00.00)
Sterilize using 0.22‐µm syringe
Aliquot and store ≤6 months at −20°C
  Store thawed aliquots at 4°C and protect from repeated freeze‐thaw cycles.

### GBM medium

500 ml DMEM, low glucose, pyruvate (Thermo Fisher, 31885‐023)
50 ml fetal bovine serum, heat‐inactivated (Thermo Fisher, A5256801)
10 ml MEM Non‐Essential Amino Acids Solution (100X) (Thermo Fisher, 11140035)
5 ml penicillin/streptomycin (10,000 U/ml, Thermo Fisher, 15140‐122)
Store ≤1 month at 4°C

## COMMENTARY

### Critical Parameters

All regulatory requirements must be fulfilled, and animal experiments must be approved by the relevant local authorities before being conducted in any facility or laboratory. Although NSG mice generally tolerate this type of minimally invasive surgical intervention (Basic Protocols 1 and 2) well, aseptic conditions should be maintained throughout the procedure whenever possible to minimize the risk of adverse events. This requires careful experimental planning, sterilization of all instruments used, and dedicated postoperative monitoring and care of the animals.

Proficiency in general mouse handling is essential for all animal experiments. In addition, successful implementation of the basic protocols requires experience and technical proficiency in operating a stereotactic frame or stereotactic robot. Such expertise not only reduces the time required to perform stereotactic injections but also minimizes animal burden while improving procedural precision, reproducibility, and overall scientific rigor.

Needle movement and injection rate are additional critical parameters. Advancement of the needle into the target region of the brain should be performed slowly and steadily to minimize acute tissue trauma. Likewise, the cell suspension should be injected at a controlled rate to prevent reflux caused by tissue counterpressure. Following injection, the needle should remain in place for ≥1 min before being withdrawn slowly and steadily to minimize efflux of the injected material.

Every effort should be made to use tumor cells of the same low passage number that have been maintained under identical culture conditions before implantation. Likewise, CAR T cells should be freshly manufactured for each experiment and thoroughly characterized *in vitro* prior to administration. Consistency in cell culture conditions, cellular fitness, and product characterization is essential for minimizing experimental variability and ensuring reproducible treatment outcomes.

### Troubleshooting

The *in vivo* growth kinetics of each tumor cell line (Basic Protocol 1) are unique; therefore, preliminary experiments to characterize tumor growth can be critical for successful study design. In our model, CAR T cells were administered (Basic Protocol 2) 7 days after tumor engraftment, based on prior characterization of the growth kinetics of the respective cell line. Selection of an inappropriate treatment initiation time point can substantially affect the observed therapeutic outcome and should therefore be considered during troubleshooting.

If treatment is initiated too late, tumors may have progressed beyond a stage at which a measurable therapeutic effect can be achieved. Conversely, treatment administered too early may not adequately reflect the efficacy of the intervention against established disease. In either case, the resulting treatment and survival data may not accurately represent the true therapeutic potential of the CAR T cell product and may therefore lack robustness and reproducibility. Careful optimization of the treatment initiation time point based on tumor growth characteristics is thus essential for generating interpretable and reliable results.

If tumors develop in an unintended anatomical location, the mouse skull may not have been secured properly in the stereotactic frame, resulting in inaccurate injections due to erroneous stereotactic coordinates. Ensure that the head is firmly immobilized and can only tilt up and down but not move in any other direction. In addition, the difference between Bregma and Lambda along the dorsoventral axis should not exceed 0.03 mm.

If tumors fail to engraft or exhibit little to no growth, the tumor cells may have been exposed to excessive mechanical stress during the procedure or may have remained in suspension on ice for an extended period of time before implantation. To assess potential cell damage, plate an aliquot of the injected cells in culture and monitor their *in vitro* growth characteristics and viability.

To minimize variability in *in vivo* BLI (Support Protocol 2), which may affect tumor visualization and quantification, proper technique for intraperitoneal injections and consistent animal handling are essential throughout anesthesia induction, imaging acquisition, and recovery. D‐luciferin should be allowed to circulate for ≥10 min before imaging to ensure adequate penetration across the blood‐brain barrier and sufficient substrate availability within intracranial xenografts. Furthermore, hypothermia should be avoided before, during, and after imaging, as it may influence signal intensity and overall animal well‐being.

### Understanding Results

With sufficient technical expertise, the intended intracranial target coordinates can be reached reliably and reproducibly. Combined with appropriately cultured tumor cells harvested during the logarithmic growth phase and properly generated CAR T cells that demonstrate robust antigen‐specific activity *in vitro*, this model (Basic Protocols 1 and 2) enables reliable assessment of therapeutic efficacy through both survival studies and endpoint analyses.

BLI and flow cytometric analysis of brain tissue (Support Protocols 1 and 2, respectively) provide complementary methods for monitoring tumor progression and quantifying treatment response. Together, these approaches allow precise evaluation of tumor regression and residual tumor burden, thereby facilitating assessment of the depth of response achieved by CAR T cell therapy.

Overall, this orthotopic GBM model provides a robust and clinically relevant platform for preclinical evaluation of CAR T cell therapies. By enabling comprehensive assessment of antitumor activity *in vivo*, it contributes to the identification and optimization of promising therapeutic strategies and supports their translation into clinical applications.

### Time Considerations

To maintain aseptic conditions as much as possible, the stereotactic frame, all surgical instruments, and the workspace should be sterilized both on the day before and immediately prior to surgery. With sufficient technical expertise and routine, the procedures for tumor implantation and intraventricular CAR T cell administration described here (Basic Protocols 1 and 2, respectively) require approximately 45 to 60 min per mouse using a single stereotactic frame.

*In vivo* BLI (Support Protocol 1), including i.p. administration of D‐luciferin, anesthesia induction, image acquisition, and recovery of the animals, requires ∼30 min for a cohort of five mice when using an IVIS imaging system equipped with five nose cones.

Depending on the experimental setup and animal facility, the time required to harvest mouse brains for flow cytometric analysis (Support Protocol 2) may vary considerably. Once the brain tissue has been collected and is ready for processing, tissue dissociation, staining, and flow cytometric acquisition typically require an additional 3 to 4 hr.

### Author Contributions

**Julian Hübner**: Conceptualization; data curation; methodology; validation; visualization; writing—original draft; writing—review and editing. **Rhonda McFleder**: Conceptualization; methodology; writing—review and editing. **Chi Wang Ip**: Methodology; resources. **Miriam Alb**: Validation; writing—review and editing. **Hermann Einsele**: Funding acquisition; resources. **Michael Hudecek**: Funding acquisition; resources. **Thomas Nerreter**: Conceptualization; funding acquisition; resources; supervision; validation; writing—review and editing.

### Conflict of Interest

M.H. is listed as the inventor on patent applications and has been granted patents related to CAR T cell technologies that have been filed by the Fred Hutchinson Cancer Research Center, Seattle, Washington, USA. These technologies have been in part licensed by industry. In addition, M.H. and T.N. are listed as inventors on patent applications and have been granted patents related to CAR T cell technologies that have been filed by the University of Würzburg, Würzburg, Germany. These technologies have also been in part licensed by industry. M.H. is a cofounder and equity owner of T‐CURX GmbH, Würzburg, Germany, and T.N. is employed at T‐CURX GmbH, Würzburg, Germany. M.H. has received honoraria from BMS, Janssen, and Kite/Gilead. The other authors declare that they have no competing interests.

## Data Availability

Data sharing is not applicable to this article, as no new data were generated.
